# Observations on the Digestive Power of the Human Stomach

**Published:** 1804-01-01

**Authors:** J. Helm


### Observations on the Digestive Power of the Human Stomach, *by* J. Helm; *communicated by* Dr. Noehden.

THE author having the care of a woman, 58 years of age, who suffered under a remarkable disease of the stomach, took the opportuuity of making several observations on the action of the stomach on different aliments. The subject of his observations had always enjoyed a good state of health, till she was married in her thirty-third year of age, when she was frequently attacked with cholics, vomitings, a redness, a burning and pungent sensation in the region of the stomach, which symptoms generally returned every third week, but were less violent in time of pregnancy. A blister, applied on the painful place, relieved her greatly, and during the space of one year, she found herself free from pain. After this time, she became pregnant, and about the middle of this state, she observed a tumour in the region of the stomach, of the size of a walnut, which had the same colour as the skin, and was painful. After the delivery, those symptoms recurred with greater violence, and were almost insupportable in rainy weather. Appetite good. The tumour began gradually to increase and to become hard till it reached down to the navel, and an anasarca supervened. Thus it remained till the next year, when the tumour being accidentally pressed, broke open, and about a pound of yellow purulent matter issued, when her sufferings diminished, together with the anasarca. The opening was of the size of a small pea, and small particles ticles of aliments came forth from it. Mr. H. being sent for about this time, found the patient weak and emaciated; the opening gradually increased, so as to let a finger pass into the stomach; a prolapsus of the opposite side of the stomach supervened, and a catheter could be introduced as far as the pylorus, without either much incommoding the patient, or causing vomition. In this state, the patient remained five years, and during this time the author made the following observations. The food of the patient was extremely simple, wine causing much pain. Some hours after her meal, she had violent pains in the back, reaching thence to the left side, and particularly a burning sensation about the edges of the opening, and she was generally obliged to loosen the bandage, and to let the contents of the stomach issue, while she relieved the pain by drinking milk. When the weather was fine, she had neither hunger nor thirst, and sometimes fasted for about thirty hours. The taking of sour krout or pickled cucumbers, sallad, and sour cherries or apples, gave her the least pain, but any flatulent food she could not keep above an hour in her stomach. In bad weather, she felt a great deal of pain, and the prolapsus increased. Any violent motion would immediately make a good deal of gall run into the stomach, from which it issued through the external opening. Milk coagulated instantly after it came into the stomach, either through the mouth or the opening, which however arrived a little later, when the stomach had been washed with water; asses milk coagulated latest of all. The patient was never obliged to bring out any spittle, nor could she be made to vomit by a mechanical irritation. Stools followed regularly every second or third day; urine was yellowish and cloudy; pulse 80—85 in a minute, and after the taking of spirits, it became 112—120 in a minute, and then the food issued with force out of the opening. A solution of one grain of emetic tartar being brought into the stomach with a syringe, caused much trouble, and a burning sensation, and the peristaltic motion of the stomach could be clearly seen. The pains were relieved by milk; and after three hours, she had four watery stools.

The results of other experiments with aliments and medicines are as follow: White bread of all sorts was more easily digestible than brown or black bread. Peas and lentiles were more digestible than beans. Pears and pine-apples more than apples. Peaches, apricots, plumbs and figs were easily digestible. Chesnuts, nuts, and sweet almonds were almost indigestible, with and without the epidermis.

epidermis. Turnips and potatoes were very digestible. Sallad and different sorts of cabbages, prepared with vinegar, were easily digestible. All sorts of champignons were difficult to digest. Veal, lamb's flesh, and pork were more easily digestible than beef, mutton, and flesh of a wild boar; not so easily as from a hare or other venison. Chickens, pigeons, &c. were more easy to digest than geese or duck's flesh. The intestines of animals were easy to digest; ham was easily digestible; all sorts of fish were soon digested. Sixty grains of Peruvian bark had lost, after having remained from six to ten hours in the stomach, ten grains; the extracts of bark, cicuta, aconitum, had lost in the same time, twenty grains; gummi guaiacum, limatura martis, and antimonium crudum, from ten to twelve grains. Castoreum, wax, assafœtida, and rusty iron filings remained insoluble; ten grains of camphor, were within six to twenty-four hours, diminished from four to six grains. Gum arabic was quite dissolved; sixty grains of g. ammoniac. lost often, in ten or twelve hours, from eighteen to twenty grains. The weight of magnesia usta was increased twenty grains in the same time, and shewed no more any effervescence with acids. Flores sulphuris alone were insoluble, but by the addition of a neutral salt they became a little soluble.

